# Lower Bounds on the Number of Realizations of Rigid Graphs

**DOI:** 10.1080/10586458.2018.1437851

**Published:** 2018-03-27

**Authors:** Georg Grasegger, Christoph Koutschan, Elias Tsigaridas

**Affiliations:** a Johann Radon Institute for Computational and Applied Mathematics (RICAM), Austrian Academy of Sciences, Linz, Austria; b Sorbonne Universités, UPMC Univ Paris 06, CNRS, INRIA, Laboratoire d’Informatique de Paris 6 (LIP6), Équipe PolSys, Paris Cedex, France

**Keywords:** Laman graph, minimally rigid graph, graph realization, 52C25, 05C04, 68W30

## Abstract

Computing the number of realizations of a minimally rigid graph is a notoriously difficult problem. Toward this goal, for graphs that are minimally rigid in the plane, we take advantage of a recently published algorithm, which is the fastest available method, although its complexity is still exponential. Combining computational results with the theory of constructing new rigid graphs by gluing, we give a new lower bound on the maximal possible number of (complex) realizations for graphs with a given number of vertices. We extend these ideas to rigid graphs in three dimensions and we derive similar lower bounds, by exploiting data from extensive Gröbner basis computations.

## 1. Introduction

The theory of rigid graphs forms a fascinating research area in the intersection of graph theory, computational (algebraic) geometry, and algorithms. Besides being a very interesting mathematical subject, rigid graphs and the underlying theory of Euclidean distance geometry have a huge number of applications ranging from robotics [Faugère and Lazard 95, Walter and Husty 07a, Walter and Husty 07b] and bioinformatics [Emiris and Mourrain 99, Jacobs et al. 01, Liberti et al. 11, Liberti et al. 14, Mucherino et al. 12] to sensor network localization [Zhu 10] and architecture [Emmerich 88]. Upper and lower bounds on the number of realizations (embeddings) of rigid graphs are of great importance as they quantify the difficulty of the problem(s) at hand that we are interested in.

We first give some definitions, to set the context of our study. Let *G* be a graph and provide to $R^{d}$ the Euclidean metric; in this way we obtain the Euclidean *d*-dimensional space. By specifying the coordinates of the vertices of *G* in $R^{d}$ we obtain a *realization*, or *embedding*, of *G* in $R^{d}$. If there is no continuous deformation of the graph that preserves the edge lengths, then the embedding is called *rigid*. A graph *G* is said to be *generically rigid* in $R^{d}$ if and only if all of its generic realizations are rigid. In the case of $R^{2}$ these graphs are also known as *Laman graphs*.

Given a generically rigid graph in $R^{d}$, together with generic edge lengths, we can embed it in the Euclidean *d*-space in a finite number of ways, modulo rigid motions (translations and rotations). It is of great interest to provide tight bounds for the number of embeddings of such graphs, modulo rigid motions, for any *d*. Our results provide lower bounds for *d* = 2 and *d* = 3.

### 1.1. Previous work

The first bounds on the number of realizations of rigid graphs, using degree bounds from algebraic geometry, are due to Borcea and Streinu [Borcea and Streinu 04]. They rely on the theory of distance matrices and on bounds of determinantal varieties. This results in the upper bounds 2n-4n-2=Θ(4n/n) for graphs in 2D, and 2n-3n-22n-6n-3=Θ(8n/(nn)) for graphs in 3D, where *n* denotes the number of vertices. Steffens and Theobald [Steffens and Theobald 10] improved these bounds by exploiting the sparsity of the underlying polynomial systems. These bounds were further improved by applying additional tricks to take advantage of the sparsity and the common sub-expressions that appear in the polynomial systems [Emiris et al. 09, Emiris et al. 13]. A direct application of the mixed volume techniques, which roughly speaking capture the sparsity of a polynomial system, yields a bound of 4^*n* − 2^ for the planar case. If we also take into account the degree of the vertices, then in the 2D case, for a Laman graph with *k* ⩾ 4 degree-2 vertices, the number of planar embeddings of *G* is bounded from above by 2^*k* − 4^4^*n* − *k*^. For the 3D case, when the graph is the 1-skeleton of a simplicial polyhedron with *k* ⩾ 9 degree-3 vertices, then the number of embeddings is bounded from above by 2^*k* − 9^8^*n* − *k*^.

The state-of-the-art result is the recent paper [Capco et al. 17b] that provides an algorithm for computing the number of complex realizations of Laman graphs. The algorithm recursively computes these numbers by lifting the problem to pairs of graphs. Arguments from tropical geometry are used to show the correctness of the algorithm, while the computations themselves are then purely combinatorial (an implementation can be found at [Capco et al. 16]). With help of the algorithm, the number of realizations of all Laman graphs up to 12 vertices were computed. We exploit these data in the present paper.

The first lower bounds for graphs in 2D were 24^⌊(*n* − 2)/4⌋^ (approx. 2.21^*n*^) and 2 · 12^⌊(*n* − 3)/3⌋^ (approx. 2.29^*n*^), that exploited a gluing process using a caterpillar, resp., fan construction [Borcea and Streinu 04], see also [Emiris et al. 09]. Both constructions use the three-prism graph (sometimes also called Desargues graph) as a building block, which is a graph with 6 vertices and 24 embeddings. More recent lower bounds are 2.30^*n*^ from [Emiris and Moroz 11] and 2.41^*n*^ from [Jackson and Owen 18]. For graphs in 3D, the only known lower bound is 16^⌊(*n* − 3)/3⌋^ (approx. 2.52^*n*^) for *n* ⩾ 9, which uses a cyclohexane caterpillar as building block [Emiris et al. 09].

### 1.2. Our contribution

We present lower bounds on the maximal number of planar, resp., spatial, embeddings (up to rigid motions) of minimally rigid graphs with a prescribed number of vertices. However, we relax the condition that the embeddings take place in $R^{d}$. Instead, we compute the number of *complex* Euclidean embeddings, that is embeddings in $C^{d}$. In this complex setting, even the edge lengths may be assumed to be complex numbers. Clearly, the number of complex embeddings is an upper bound on the number of real embeddings.

Using the novel algorithm developed in [Capco et al. 17b], we compute the exact number of planar embeddings for graphs with a relatively small number of vertices. In contrast, the number of spatial embeddings is computed probabilistically by means of Gröbner bases. Then we introduce techniques to “glue” an arbitrary number of such small graphs in order to produce graphs with a high number of vertices (and edges) that preserve rigidity. The gluing process (see Sections 2.1 and 3.1) allows us to derive the number of embeddings of the final graph from the number of embeddings of its components, and in this way we derive a lower bound for the number of embeddings in $C^{2}$ (Theorem 5) and in $C^{3}$ (Theorem 7). We emphasize that the gluing techniques are quite general and can be extended to arbitrary dimensions. Moreover, to identify those small graphs that realize the maximum number of embeddings and that can be the building blocks for the gluing process, we perform extensive experiments. We use the state-of-the-art computer algebra tools to count the number of embeddings as the maximum number of complex solutions of polynomial systems.

If we were able to compute the number of embeddings of the small graphs in $R^{d}$, for example, by using the approach proposed in [Emiris and Moroz 11], then we could transfer our lower bounds on the complex embeddings to the number of real embeddings, by applying the very same gluing process; see also [Jackson and Owen 18] for gluing processes using the caterpillar graph. There it is also hinted that the numbers of real and complex embeddings do not match in general. It is a very interesting problem to quantify this gap. On the one hand, one can construct infinite families of graphs for which the ratio between real and complex embeddings tends to zero. On the other hand, there are graphs, see [Emiris and Moroz 11] for a nontrivial example, where edge lengths can be found such that there exist as many real embeddings as complex ones.

### 1.3. Organization of the paper

The paper is structured as follows: First (Section 2), we present the construction of the lower bounds for the planar case, and in Section 3, we present the lower bounds for the spatial case. In Section 2.1, we describe three constructions (gluing processes) for producing infinite families of rigid graphs. Then, in Section 2.2, we discuss several strategies to identify expedient graphs that are suitable for these constructions. They lead to new lower bounds, which is discussed in Section 2.3

Throughout the paper, we represent a graph by the integer obtained by flattening the upper triangular part of its adjacency matrix and interpreting this binary sequence as an integer. For further details, we refer to the Appendix. There we also collect the encodings of all graphs mentioned throughout the paper.

## 2. Dimension 2

We begin our study with the case of planar embeddings. For this purpose, we recall the definitions of some fundamental notions. The goal in this section is to derive lower bounds for the quantity *M*_2_(*n*), introduced in Definition 2 below.

Definition 1. A *Laman graph* [Laman 70] is a graph *G* = (*V*, *E*) such that |*E*| = 2|*V*| − 3, and such that |*E*′| ⩽ 2|*V*′| − 3 holds for every subgraph *G*′ = (*V*′, *E*′) of *G*.

Definition 2. For a Laman graph *G* = (*V*, *E*), we define Lam_2_(G), called the *Laman number* of *G*, to be the number of (complex) planar embeddings that a generic labeling λ:E→C (the “edge lengths” of *G*) admits. Moreover, we define *M*_2_(*n*) to be the largest Laman number that is achieved among all Laman graphs with *n* vertices.

**Figure 1.**
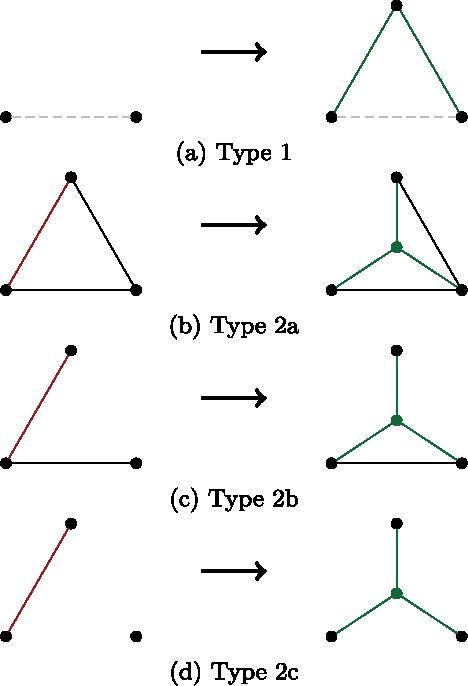
Henneberg steps of different types in dimension 2; A dashed line indicates that this edge can exist but does not need to.

In [Henneberg 03], Laman graphs are characterized to be constructible from a single edge by a sequence of two types of steps (see Figure 1). We call them Henneberg steps of type 1 and type 2, respectively. The steps of type 2 can be further classified according to additional occurring edges.

**Figure 2.**
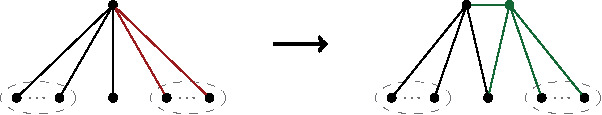
Vertex splitting.

It is well known that a Henneberg step of type 1 always increases the Laman number by a factor of 2. So far it is not known by which factor a Henneberg step of type 2 might increase the Laman number. As mentioned in [Jackson and Owen 18], there are Henneberg steps of type 2 which do increase the Laman number by a factor of less than 2. Vertex splitting is another construction preserving rigidity (see Figure 2). In [Jackson and Owen 18], it is shown that vertex splitting increases the Laman number by a factor of at least two. Henneberg steps of type 2a and 2b are special cases of vertex splitting. Hence, only type 2c can yield a factor of less than two. Table 1 shows some increases of Laman numbers, given a certain Laman graph *G* and constructing a new one *G*′ by a single Henneberg step.

**Table 1.** Henneberg constructions and increase of Laman numbers.

Type	*G*	Lam_2_(G)	*G*′	Lam_2_(G′)	Factor
2c	1269995	56	31004235	96	1.71
2c	7916	24	481867	44	1.83
2b	186013	32	170989214	136	4.25
2c	183548	32	170989214	136	4.25
2c	20042142	64	11177989553	344	5.37
2c	4593214614	128	22301628505804	808	6.31
2c	1248809223262	256	2960334732174949	1976	7.72
2c	1710909647295913	512	15006592507478215906	4816	9.41

### 2.1. Constructions

We discuss different constructions of infinite families of Laman graphs ${\left(G_{n}\right)}_{n\in N}$ with *G_n_* having *n* vertices. We do this in a way such that we know precisely the Laman number for each member of the family. This directly leads to a lower bound on *M*_2_(*n*). The ideas of these constructions are described in [Borcea and Streinu 04]; they were used to get lower bounds by connecting several three-prism graphs at a common basis. Here, we generalize them in order to connect any Laman graphs at an arbitrary Laman base. We present three such constructions.

#### 2.1.1. Caterpillar construction

**Figure 3.**
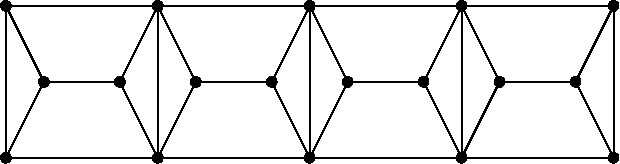
Caterpillar construction with four copies of the three-prism graph.

The “caterpillar construction” [Borcea and Streinu 04] works as follows: place *k* copies of a Laman graph *G* = (*V*, *E*) in a row and connect every two neighboring ones by means of a shared edge (see Figure 3). Alternatively, one can let all *k* graphs share the same edge. In any case, the resulting assembly has 2 + *k*(|*V*| − 2) vertices and its Laman number is Lam_2_(G)^k^, since each of the *k* copies of *G* can achieve all its Lam_2_(G) different embeddings, independently of what happens with the other copies. Hence, among all Laman graphs with *n* = 2 + *k*(|*V*| − 2) vertices there exists one with Lam_2_(G)^k^ embeddings. If the number of vertices *n* is not of the form 2 + *k*(|*V*| − 2), then we can use the previous caterpillar graph with ⌊(*n* − 2)/(|*V*| − 2)⌋ copies of *G* and perform some Henneberg steps of type 1; as we mentioned earlier, each of these steps doubles the Laman number. Summarizing, for any Laman graph *G*, we obtain the following lower bound from the caterpillar construction:

(1) M2(n)⩾2(n-2)mod(|V|-2)· Lam
2(G)⌊(n-2)/(|V|-2)⌋(n⩾2).

#### 2.1.2. Fan construction

**Figure 4.**
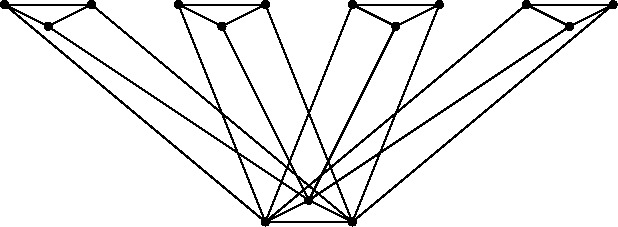
Fan construction with four copies of the three-prism graph.

The second construction we employ is called “fan construction”: take a Laman graph *G* = (*V*, *E*) that contains a triangle (i.e., a 3-cycle), and glue *k* copies of *G* along that triangle (see Figure 4). Once we fix one of the two possible embeddings of that triangle, each copy of *G* admits Lam_2_(G)/2 embeddings. The remaining Lam_2_(G)/2 embeddings are obtained by mirroring, i.e., by using the second embedding of the common triangle. Similarly as before, the assembled fan is a Laman graph with 3 + *k*(|*V*| − 3) vertices that admits 2 · (Lam_2_(G)/2)^k^ embeddings. Hence, we get the following lower bound:

(2) M2(n)⩾2(n-3)mod(|V|-3)·2· Lam
2(G)2⌊(n-3)/(|V|-3)⌋(n⩾3).

While the caterpillar construction can be done with any Laman graph, this is not the case with the fan. For example, the Laman graph with 12 vertices displayed in Figure 6 has no 3-cycle and therefore cannot be used for the fan construction (see also Table 4).

#### 2.1.3. Generalized fan construction

As a third construction, we propose a generalization of the fan construction: instead of a triangle, we may use any Laman subgraph *H* = (*W*, *F*) of *G* for gluing. Using *k* copies of *G*, we end up with a fan consisting of |*W*| + *k*(|*V*| − |*W*|) vertices and Laman number at least  Lam
2(H)·(
Lam
2(G)/
Lam
2(H))k. Here, we assume that the embeddings of *G* are divided into *L*(*H*) equivalence classes of equal size, by considering two embeddings of *G* as equivalent if the induced embeddings of *H* are equal (up to rotations and translations). If this assumption was violated, the resulting lower bound would be even better; thus we can safely state the following bound:

(3) M2(n)⩾2(n-|W|)mod(|V|-|W|)·
Lam
2(H)· Lam
2(G)
Lam
2(H)⌊(n-|W|)/(|V|-|W|)⌋(n⩾|W|).

**Figure 5.**
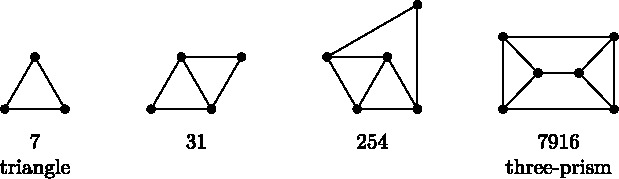
Bases for the generalized fan construction and their encodings.

Note that the previously described fan construction is a special instance of the generalized fan, by taking as the subgraph *H* a triangle with Lam_2_(H) = 2. Similarly, also the caterpillar construction can be seen as a special case, by taking for *H* a graph with two vertices and Laman number 1. To indicate the subgraph of a generalized fan construction, we also write *H*-fan. Using our encoding for graphs the usual fan would be denoted by 7-fan. The fan fixing the four-vertex Laman graph is then denoted by 31-fan. Figure 5 shows these bases.

### 2.2. Rigid graphs with many embeddings

**Figure 6.**
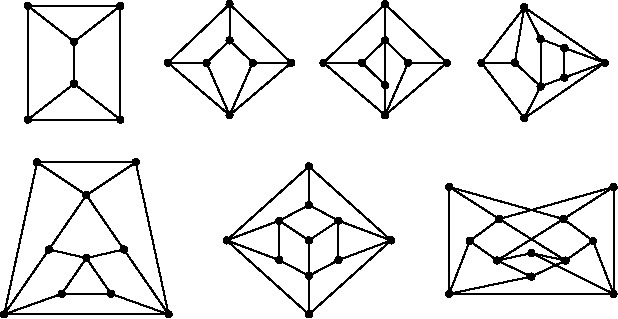
Unique Laman graphs with 6 ⩽ *n* ⩽ 12 with maximal number of embeddings (see Table 2, encodings see Table 8).

In order to get good lower bounds, we need particular Laman graphs that have a large number of embeddings. For this purpose, we have computed the Laman numbers of all Laman graphs with up to *n* = 12 vertices. We did so using the algorithm of [Capco et al. 17b] (see [Capco et al. 16] for an implementation and [Capco et al. 17a] for a streamlined extended abstract). For each 3 ⩽ *n* ⩽ 12, we have identified the (unique) Laman graph with the highest number of embeddings. We present these numbers in Table 2 and the[Table t0003 t0004] corresponding graphs for 6 ⩽ *n* ⩽ 12 appear in Figure 6.

**Table 2.** Minimal and maximal Laman number among all *n*-vertex Laman graphs; the minimum is 2^*n* − 2^ and it is achieved, for example, on Laman graphs that are constructible by using only Henneberg steps of type 1. The row labeled with “lower” contains the bounds from [Emiris et al. 09].

*n*	6	7	8	9	10	11	12
min	16	32	64	128	256	512	1024
*M*_2_(*n*)	24	56	136	344	880	2288	6180
lower	24	48	96	288	576	—	—

There are 44,176,717 Laman graphs with 12 vertices, and therefore it was a major undertaking to compute the Laman numbers of all of them; it took 56 processor days to complete this task. Hence, it is unrealistic to do the same for all Laman graphs with 13 or more vertices. In order to proceed further, we developed some heuristics to construct graphs with very high Laman numbers, albeit not necessarily the highest one. The properties that we formulate for the families *T*(*n*) and *S*(*n*) below are inspired by inspecting the few known graphs that achieve the maximal Laman number *M*_2_(*n*) (see Figure 6 and Table 2). More precisely, we consider the set *T*(*n*) of Laman graphs with *n* vertices that satisfy the following additional properties.

Definition 3. We say that a Laman graph *G* = (*V*, *E*) with *n* vertices is an element of *T*(*n*) iff

- *G* is a planar graph, that is, it can be embedded in the plane without crossings of edges.
- Each vertex of *G* has degree 3 or 4; in this case the Laman condition (Definition 1) implies that there are exactly 6 vertices of degree 3 and |V|-6 vertices of degree 4.
- There are precisely two 3-cycles, and the number of 4 cycles is |V|-3. Note that we count only nontrivial cycles all of whose edges are distinct. Moreover, the 3-cycles are disjoint, that is, they do not share an edge. By Euler's formula the number of faces (including the outer, unlimited one) is given by 2-|V|+|E|=|V|-1, and hence each of the cycles is the boundary of a face.

These properties are quite selective: for example, the set *T*(12) contains only 18 (out of 44 million!) Laman graphs, and the set *T*(18) has the manageable cardinality 188. For *n* ⩽ 11 we have that maxG∈T(n) Lam
2(G)=M2(n). In contrast, the 12-vertex graph with the highest Laman number is not in *T*(12), since it is not planar and does not have any 3-cycles. Nevertheless, it satisfies the condition on the vertex degrees. Furthermore, the graph with the highest Laman number in *T*(12) is the graph with the second highest Laman number with 12 vertices. Hence, it is also the graph with the highest Laman number which does contain a 3-cycle. For 13 ⩽ *n* ⩽ 18, we have constructed all Laman graphs *T*(*n*) and among them identified the one with the highest Laman number. We summarize the results in Table 3; the corresponding graphs are displayed in Figure 6.

**Table 3. t0003:** With *M_T_*(*n*) we denote the maximal Laman number of the graphs in *T*(*n*). In the row below we give the highest Laman numbers that we have found so far by looking at graphs in *S*(*n*) (exhaustive for *n* ⩽ 15 but incomplete for *n* > 15).

*n*	12	13	14	15	16	17	18
*M_T_*(*n*)	5952	15,056	39,696	105,384	277,864	731,336	1,953,816
*M_S_*(*n*)	6180	15,536	42,780	112,752	312,636	870,414	2,237,312

We have seen that for 12 vertices the maximal graph in *T*(12) is not the one with the highest Laman number. The same holds true for 13 ⩽ *n* ⩽ 18, which can be seen by looking at a different family of graphs: We observed that the graphs which are known to be maximal according to their Laman number are Hamiltonian, i.e., they contain a path that visits each vertex exactly once (Hamiltonian path). Hence, we focus on Hamiltonian graphs. The problem is that they cover still around 2/3 of all Laman graphs (at least for small *n*). Therefore, we considered other properties of the known graphs with maximal Laman number. One of these properties is the symmetry of a certain embedding.

Definition 4. We say that a Laman graph *G* = (*V*, *E*) is an element of *S*(*n*) iff

- *G* is Hamiltonian, i.e., it contains a Hamiltonian cycle *H*.
- There exists a circular embedding, i.e., an embedding ρ such that ρ(*v*) lies on the same circle for all *v* ∈ *V*, and *H* is embedded on a regular *n*-gon.
- The figure obtained by the embedding is point, resp., line symmetric for an even, resp., odd number of vertices.

One can see that the maximal Laman graphs up to 12 vertices fulfill these symmetry properties (Figure 8).

We computed the Laman numbers of all graphs in *S*(*n*) up to *n* = 15. Unfortunately, for larger *n* the set *S*(*n*) still contains too many graphs. For *n* = 15 there are already 85,058 such graphs. Performing the computations on a subset of *S*(*n*) yields the graphs shown in Figure 9.

### 2.3. Lower bounds

We now use these results to derive new and better lower bounds than the previously known ones. We apply the caterpillar construction to the Laman graphs with the maximal number of embeddings for 6 ⩽ *n* ⩽ 12, and for 13 ⩽ *n* ⩽ 18 we use the graphs found by exploring the set *S*(*n*) (see Figure 9 and Table 3). The fan construction is applied to the maximal Laman graphs for 6 ⩽ *n* ⩽ 11 only, since it is not applicable to the maximal graph with 12 vertices (Figure 6). Hence, for the remaining cases, 12 ⩽ *n* ⩽ 18, the fan construction is applied to the maximal graph in *T*(*n*). In Table 4, the results obtained by these graphs are written in a separate column. The results in the next column are obtained by randomly found graphs which contain a triangle and have a higher Laman number than the one in *T*(*n*).

**Table 4. t0004:** Growth rates (rounded) of the lower bounds. For *n* ⩽ 12 these values are proven to be the best achievable ones; for *n* > 12 the values are just the best we found by experiments, hence it is possible that there are better ones. The drawings of the graphs corresponding to the last three columns are given in Figure 5. The encodings for the graphs can be found at: caterpillar (Table 8), fan *T*(*n*) (Table 9), fan (Table 11), 31-fan (Table 12), 254-fan (Table 13), 223-fan and 239-fan (Table 14), 7916-fan (Table 15).

*n*	caterpillar	fan *T*(*n*)	fan	31-fan	254-fan	7916-fan
6	2.21336	2.28943		2	2	—
7	2.23685	2.30033		2.28943	2	2
8	2.26772	2.32542		2.30033	2.28943	2
9	2.30338	2.35824		2.35216	2.30033	2.28943
10	2.33378	2.38581		2.35824	2.35216	2.30033
11	2.36196	2.41159		2.38581	2.35824	2.35216
12	2.39386	2.43198		2.43006	2.39802	2.35824
13	2.40453	2.44156	2.44498	2.44772	2.42197	2.39802
14	2.43185	2.45868	2.46087	2.46391	2.44251	2.42197
15	2.44695	2.47445		2.47076	2.45031	2.42906
16	2.46890	2.48657		2.48794	2.47166	2.43712
17	2.48875	2.49668	2.49779	2.49160	2.48043	2.46341
18	2.49378	2.50798				

For 7 ⩽ *n* ⩽ 11, we also tried the generalized fan construction: among all Laman graphs whose vertex degrees are at least 3—we can exclude Laman graphs that have vertices of degree 2 since they can be derived from a smaller graph by Henneberg steps of type 1, thereby only doubling the embedding number—we selected all graphs that have the four-vertex Laman graph as a subgraph. Then we computed their Laman numbers in order to find the maximum that can be achieved among those graphs. Until 12 vertices the lower bounds, according to (3), are not as good as those obtained by the standard fan construction. For higher *n*, randomly found graphs show improvements over the fan construction for the graphs we have found. Note that since the graphs are found only randomly this does not show any results on whether the factors are indeed better.

**Figure 7.**
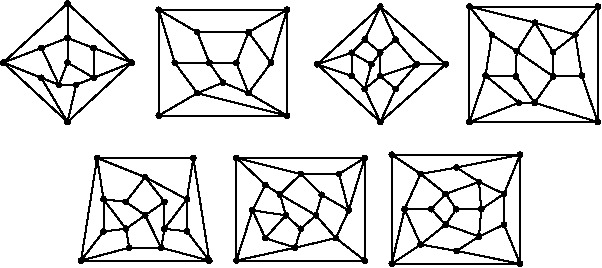
Laman graphs in *T*(*n*) with 12 ⩽ *n* ⩽ 18 vertices; for each *n* the graph with the largest Laman number among the Laman graphs in *T*(*n*) is displayed. The corresponding Laman numbers are given in Table 3 (encodings see Table 9).

From Table 4 (Figure 11), we can see the bound 2.28943^*n*^ obtained in [Borcea and Streinu 04], 2.30033^*n*^ from [Emiris and Moroz 11], and 2.41159^*n*^ from [Jackson and Owen 18], as well as the current improvements obtained in this paper. By instantiating Formula (2) with the last Laman graph in Figure 7, which has 18 vertices and Laman number 1953816, we obtain the following theorem.

**Figure 8.**
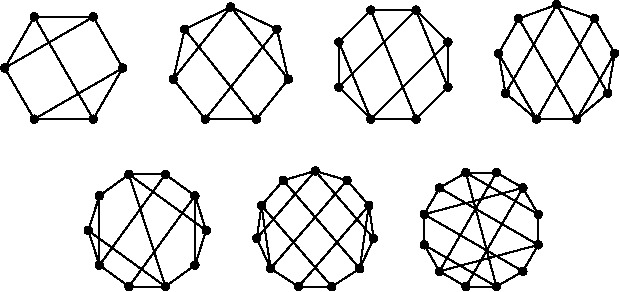
Circular embedding of the Laman graphs with maximal Laman numbers for 6 ⩽ *n* ⩽ 12. Note that these are the same graphs that are displayed in Figure 6.

**Figure 9.**
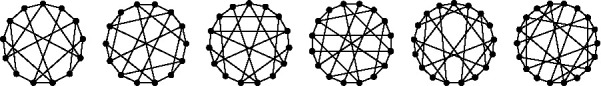
For *n* = 13, …, 18, we display the graph from *S*(*n*) with the highest Laman number (given in Table 3) found so far (encodings see Table 10).

**Figure 10.**
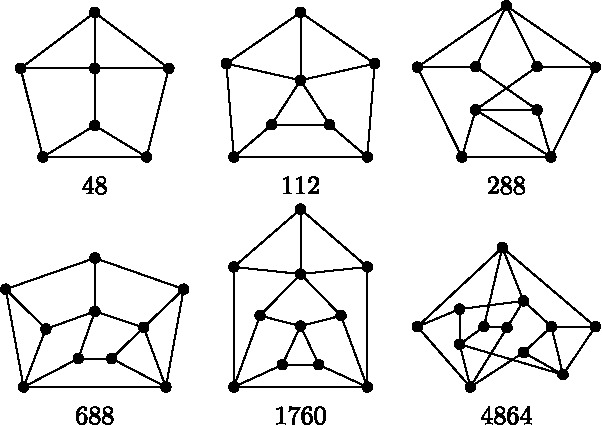
Laman graphs with 7 ⩽ *n* ⩽ 12 vertices that have the four-vertex Laman graph (encoded as 31) as a subgraph; below their Laman numbers are given. In some cases, there are several Laman graphs with this subgraph property and with the same Laman number, but among all Laman graphs that have this subgraph there does not exist one with higher Laman number (encodings see Table 12).

**Figure 11.**
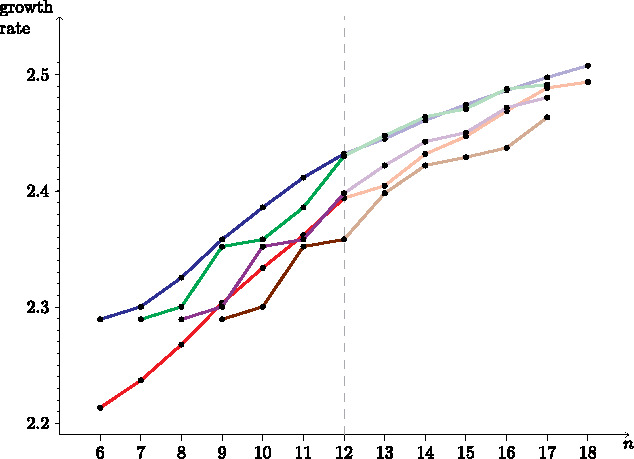
Growth rates of the lower bounds (red = caterpillar construction, blue = fan construction, green = 31-fan construction, fuchsia = 254-fan construction, brown = 7916-fan construction). The light colors indicate values that were not found by exhaustive search and which therefore could possibly be improved.

Theorem 5. The maximal Laman number *M*_2_(*n*) satisfies

M2(n)⩾2·2(n-3)
mod
15·976908⌊(n-3)/15⌋.

This means *M*_2_(*n*) grows at least as $(\;\sqrt[{15}]{976908}{)}^{n}$, which is approximately 2.50798^*n*^. In other words $(\;\sqrt[{15}]{976908}{)}^{n}\in O\left(M_{2}\left(n\right)\right)$.

## 3. Dimension 3

A generalization of the counting condition to three dimensions would suggest that a graph *G* = (*V*, *E*) needs to fulfill |*E*| = 3|*V*| − 6, and |*E*′| ⩽ 3|*V*′| − 6 for every subgraph *G*′ = (*V*′, *E*′) of *G* in order to be rigid. Unlike the two-dimensional case, this definition is necessary but not sufficient for generic minimal rigidity. An example of a graph which is not minimally rigid in dimension 3 can already be found in [Pollaczek-Geiringer 27]. We are interested in lower bounds on *M*_3_(*n*), which is the three-dimensional analog of *M*_2_(*n*).

Definition 6. Let *G* = (*V*, *E*) be a graph. We call *G* a Geiringer graph,^1^1As Hilda Pollaczek-Geiringer had already worked on rigid graphs in 2D and 3D [Pollaczek-Geiringer 27, Pollaczek-Geiringer 32], long before Gerard Laman [Laman 70]. if there exists only a finite number of (complex) spatial embeddings in $C^{3}$, given a generic labeling λ:E→C of the edges of *G*.

For a Geiringer graph *G*, we define Lam_3_(G), called the *3D-Laman number* of *G*, to be this finite number of (complex) embeddings. Moreover, we define *M*_3_(*n*) to be the largest 3D-Laman number that is achieved among all Geiringer graphs with *n* vertices.

**Figure 12.**
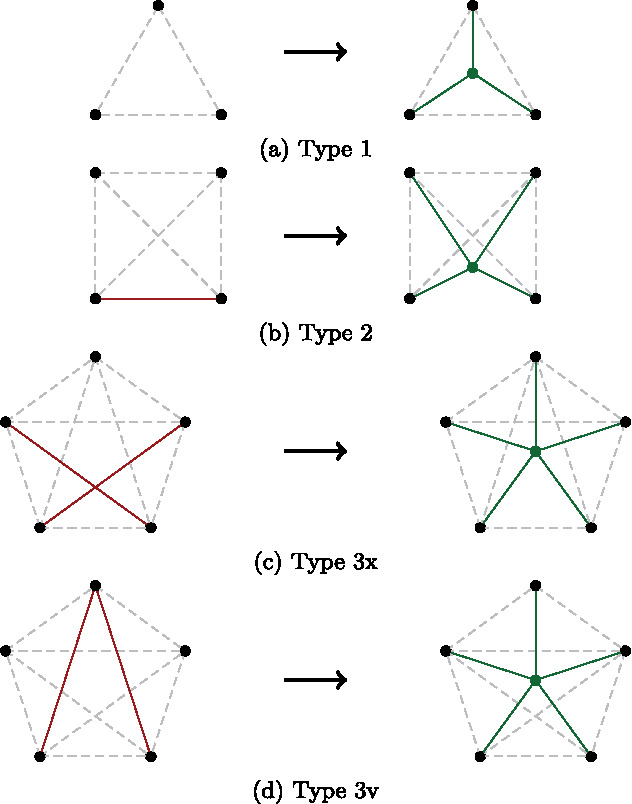
Henneberg steps of different types in dimension 3; a dashed line indicates that this edge can exist but does not need to.

**Figure 13.**
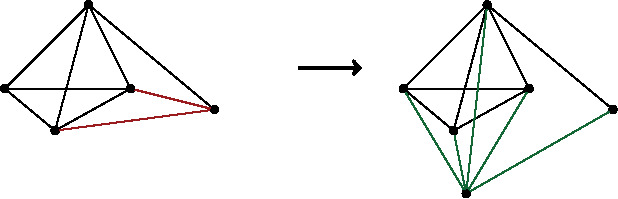
Flexible graph constructed by a Henneberg move of type 3.

In [Tay and Whiteley 85], Geiringer graphs are shown to be constructible from a triangle graph by a sequence of three types of steps (see Figure 12). Steps of type 1 and type 2 preserve rigidity (see [Tay and Whiteley 85]). The steps of type 3 can be further classified according to whether the two chosen edges have a common vertex or not. Note that every Geiringer graph can be constructed using such steps [Tay and Whiteley 85, Prop. 4.1, 4.4, 4.5], but not every construction by these steps is indeed minimally rigid, i.e., rigidity is not necessarily preserved by steps of type 3. Indeed type 3v does not even preserve the vertex-edge-count for subgraphs (see Figure 13). However, there are certain subclasses of type 3 steps for which rigidity is preserved (see, for instance, [Tay and Whiteley 85, Graver et al. 93, Cruickshank 14]).

In the following, we construct Geiringer graphs by the above-mentioned moves, removing those which turn out to be non-rigid. By this procedure, we get all Geiringer graphs with up to 10 vertices. The computation of the number of realizations is done by Gröbner bases: The coordinates of the vertices are obtained as the solutions of a system of (quadratic) polynomial equations. Instead of keeping the edge lengths generic (by introducing a symbolic parameter for each edge), we insert random numbers (integers) for the edge lengths. Otherwise the computation would not be feasible at all. Moreover, for further speed-up, we compute the Gröbner basis only modulo a sufficiently large prime number *p* so that the occurrence of large rational numbers is avoided. In other words, the Gröbner basis computation takes place over the finite field $Z_{p}$. In order to get high confidence into the results, we did each computation at least three times, with different random choices of the parameters. If we get the same result three times, we can be rather sure to have the correct number. However, we want to make the reader aware of the fact, that it is a probabilistic method. Although we have a strong evidence for the computed 3D-Laman numbers, they are not rigorously proven to be correct.

Still, computing the 3D-Laman numbers for all Geiringer graphs of 10 vertices was a major undertaking. By applying the Henneberg steps depicted in Figure 12 in all possible ways, we obtained 747,065 graphs that potentially had the property of being minimally rigid (our Gröbner basis computations suggested that 612,884 of them indeed have this property). In our implementation, we do some preprocessing on the graphs in order to create polynomial systems with as few variables as possible: for example, we remove vertices of valency 3 (i.e., revert Henneberg steps of type 1), and compensate by multiplying the final Laman number by 2 for each removed vertex. Another optimization consists in identifying the largest tetrahedral subgraph, i.e., the largest subgraph that can be constructed by Henneberg steps of type 1, starting from a triangle. This subgraph is considered when fixing some vertices of the graph, in order to deal with rotations and translations. Then we call the fast FGb [Faugère 10] implementation of Gröbner bases in Maple, for determining the number of solutions of the constructed polynomial system. Executing this program once for all 747,065 graphs took about 162 days of CPU time, using Xeon E5-2630v3 Haswell 2,4Ghz CPUs. However, the computations were run in parallel so that the result was obtained after a few days. This means that in average it took about 19 s to determine the 3D-Laman number of a graph with 10 vertices, but the timings vary a lot: graphs which can be constructed by Henneberg steps of type 1 require almost no time, due to our preprocessing, while the Gröbner basis computation for some graphs takes several hours (up to 16 hours).

It is easy to see that a Henneberg step of type 1 always increases the 3D-Laman number by a factor of 2. So far it is not known by which factor a Henneberg step of type 2 or type 3 might increase the 3D-Laman number. Table 5 summarizes some increases of 3D-Laman numbers, given a certain Geiringer graph *G* and constructing a new one *G*′ by a single Henneberg step.

**Table 5.** Henneberg constructions and increase of 3D-Laman numbers.

Type	*G*	Lam_3_(G)	*G*′	Lam_3_(G′)	Factor
3v	11717490611	512	9634462543324	128	0.25
3v	49724126	160	18848282483	64	0.40
3v	515806	48	203906043	32	0.66
2	981215	24	31965132	24	1.00
3x	16350	16	1973983	16	1.00
2, 3x	1973983	16	49524604	128	8.00
3x	384510	16	49724126	160	10.00
3v	382463	16	49724126	160	10.00
3x	15661790	32	7309884067	512	16.00
3x	2000476603	48	2704137746603	1088	22.66

### 3.1. Constructions

We consider again caterpillar and fan constructions. For the caterpillar we now need to glue two graphs by a common triangle. Similarly, we need a tetrahedron for the fan construction. For the generalized fan construction, we use the unique Geiringer graph with five vertices. For sake of completeness, we display the general formula for obtaining a lower bound on *M*_3_(*n*) with the generalized 3D-fan construction; the formula is completely analogous to (3):

(4) M3(n)⩾2(n-|W|)mod(|V|-|W|)·
Lam
3(H)·
Lam
3(G)
Lam
3(H)⌊(n-|W|)/(|V|-|W|)⌋(n⩾|W|).

### 3.2. Lower bounds for *M*_3_(*n*)

**Figure 14.**
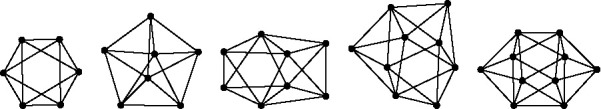
Geiringer graphs with 6 ⩽ *n* ⩽ 10 vertices; for each *n* the (unique) graph with maximal number of embeddings is depicted. The corresponding 3D-Laman numbers *M*_3_(*n*) are given in Table 6 (encodings see Table 16).

In order[Table t0006 t0007]to get good lower bounds, we need particular Geiringer graphs that have a large number of embeddings. We computed the 3D-Laman numbers of all Geiringer graphs with up to *n* = 10 vertices. For each *n*, we have identified the (unique) Geiringer graph with the highest number of embeddings. These numbers are given in Table 6. The corresponding graphs for 6 ⩽ *n* ⩽ 10 are shown in Figure 14.

**Figure 15.**
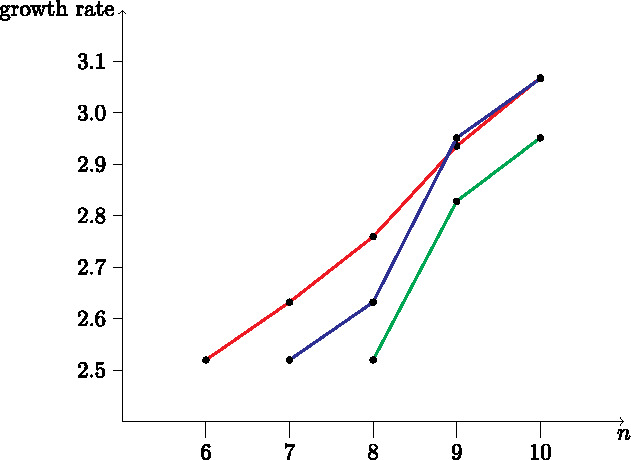
Growth rates of the lower bounds (red = caterpillar construction, blue = fan construction, green = generalized fan construction).

**Table 6. t0006:** Minimal and maximal 3D-Laman number among all *n*-vertex Geiringer graphs; the row labeled with “min” contains the lowest 3D-Laman number which is found by computation. The row labeled with “upper” contains the bounds from [Borcea and Streinu 04].

*n*	6	7	8	9	10	11	12
min	8	16	24	48	76		
*M*_3_(*n*)	16	48	160	640	2560		
upper	40	224	1344	8448	54,912	366,080	2,489,344

In [Emiris et al. 09], lower and upper bounds for the 1-skeleta of simplicial polyhedra are computed. They also use an extension of Henneberg steps to the three-dimensional case. However, they form just a subset of the Henneberg steps presented here. From Table 7 (Figure 11), we can see the bound of 2.51984^*n*^ obtained in [Emiris et al. 09] and the improvements obtained in this paper. By instantiating Formula (4) with the last Laman graph in Figure 14, which has 10 vertices and 3D-Laman number 2560, we obtain the following theorem.

**Table 7. t0007:** Growth rates (rounded) of the lower bounds. The encodings for the graphs can be found at: caterpillar (Table 16), fan (Table 17), generalized fan (Table 18).

*n*	caterpillar	fan	generalized fan
6	2.51984	2	—
7	2.63215	2.51984	2
8	2.75946	2.63215	2.51984
9	2.93560	2.95155	2.82843
10	3.06825	3.06681	2.95155

**Table 8. t0008:** Graph encodings for the graphs with maximal Laman number (see Figure 6).

*n*	Graph encoding	Laman number
6	7916	24
7	1269995	56
8	170989214	136
9	11177989553	344
10	4778440734593	880
11	18120782205838348	2288
12	252590061719913632	6180

**Table 9. t0009:** Graph encodings for the graphs in *T*(*n*) (see Figure 7).

*n*	Graph encoding	Laman number
12	757486969329934592	5952
13	3102079810848683155456	15,056
14	12393113433401056197689344	39,696
15	101535867160732294622504828928	105,384
16	283980994531838217547205604229120	277,864
17	65135173642079980743135145171586662400	731,336
18	9061092056503516236392931137633162134437921	1,953,816

**Table 10. t0010:** Graph encodings for the graphs in *S*(*n*) from Figure 9.

*n*	Graph encoding	Laman number
13	2731597771584836257824	15,536
14	3932631430916370534240769	42,780
15	94091005932357252120217796609	112,752
16	892527555716690691964688718172672	312,636
17	97035633928660816927022803757023440896	870,414
18	1132478330239973528711451061872988363235584	2,237,312

**Table 11. t0011:** Graph encodings for graphs which contain the triangle as subgraph and have high Laman number.

*n*	Graph encoding	Laman number
13	517844367551685511200	15,268
14	8465213527269428904345612	40,088
17	34561064106536153162036856640676376576	1,953,816

**Table 12. t0012:** Graph encodings for the graphs from Figure 10 and further graphs which contain the 4-vertex Laman graph as subgraph and have high Laman number.

*n*	Graph encoding	Laman number
7	127575	48
8	7654183	112
9	11987422577	288
10	26665598300033	688
11	18226243755613920	1760
12	57080320167818985484	4864
13	1845359412452332949520	12,616
14	2116433716010931973523488	32,984
15	366442648507105101448244891666	83,792
16	1054776952932226148552313881544736	224,976
17	260539761471154896904085679883542331426	570,544

**Table 13. t0013:** Laman graphs which have the 5-vertex graph with encoding 254 as subgraph.

*n*	Graph encoding	Laman number
6	3326	16
7	190686	32
8	210799326	96
9	27047004894	224
10	220302198846	576
11	511412109882689	1376
12	270814819769185025	3648
13	2585030414085585133728	9472
14	6356539347198988132306956	24,752
15	1109200018557493535348018405392	62,416
16	5598668013338146547621855406197248	168,256
17	176789006904155934327358957938973624416	433,920

**Table 14. t0014:** Laman graphs which have the 5-vertex graph with encoding 223 and 239 as subgraph, respectively.

*n*	Graph encoding	Laman number	Graph encoding	Laman number
6	12511	16	10479	16
7	111335	32	103805	32
8	6419031	96	12339295	96
9	812960551	224	1024072271	224
10	209151514913	576	221350536519	576
11	110640260854593	1376	18441562579184833	1376
12	37616617704925531361	3648	21047011153048344071	3648

**Table 15. t0015:** Laman graphs which have the three-prism with encoding 7916 as subgraph.

*n*	Graph encoding	Laman number
7	120478	48
8	6475132	96
9	51946608057	288
10	18284890201676	672
11	5366995734673421	1728
12	523614257391638273	4128
13	2066305871268252766241	10,944
14	40197303758420411293510144	28,416
15	61903368089062917457613881376	70,656
16	11358585136343922383033065301099552	177,408
17	33233417861308024077754506274593047824	486,528

**Table 16. t0016:** Graph encodings for the Geiringer graphs with maximal 3D-Laman number (see Figure 14).

*n*	Graph encoding	3D-Laman number
4	63	2
5	511	4
6	16350	16
7	515806	48
8	49724126	160
9	7345971057	640
10	3559487592083	2560

**Table 17. t0017:** Graph encodings for the Geiringer graphs which contain the tetrahedron.

*n*	Graph encoding	3D-Laman number
5	511	4
6	7679	8
7	257911	32
8	16559991	96
9	4076665507	448
10	4894450217603	1664

**Table 18. t0018:** Graph encodings for the Geiringer graphs which contain the double tetrahedron.

*n*	Graph encoding	3D-Laman number
6	7679	8
7	237055	16
8	14937975	64
9	38164887119	256
10	3168405805643	896

Theorem 7. The maximal Laman number *M*_3_(*n*) satisfies

$$M_{3}\left(n\right)⩾2^{(n-3)\;\;\;\;\;\;\;\mathrm{mod}\;7}\cdot 2560^{\left\lfloor (n-3)/7\right\rfloor }.$$

This means *M*_3_(*n*) grows at least as $(\;\sqrt[{7}]{2560}{)}^{n}$ which is approximately 3.06825^*n*^. In other words $(\;\sqrt[{7}]{2560}{)}^{n}\in O\left(M_{3}\left(n\right)\right)$.

## 4. Conclusion

By exploiting state-of-the-art methods, we gave some new bounds on the maximal possible number of realizations of rigid graphs for a given number of vertices. Further systematic computations would exceed reasonable time constraints. The results obtained by our analysis give of course rise to further research. It is still an open problem how graphs which have the maximal number of realizations can be classified, and how to bound this number:

Open Problem 1. Find an upper bound *b_n_* < 4^*n* − 2^ such that Lam_2_(G) ⩽ b_n_ for all Laman graphs *G* with *n* vertices.

From our data, we observe that (for *n* ⩽ 12) there is always a unique graph *G*_*n*, max _ on *n* vertices that achieves the maximal Laman number among all graphs with *n* vertices.

Conjecture 2. For each *n* ⩾ 2, there is a unique Laman graph *G*_*n*, max _ with *n* vertices and with the property Lam_2_(G_n, max _) = M_2_(n). Similarly for *M*_3_(*n*).

Also the relation of Henneberg steps to the increase of the number of realizations is subject of further research:

Open Problem 3. Find lower and upper bounds for the factor $\mathrm{Lam}\left(G^{'}\right)/\mathrm{Lam}\left(G\right)$ where *G*′ is constructed for a Laman graph *G* by a Henneberg step. By what we showed, the lower bound is smaller than or equal to 12/7 in 2D and 1/4 in 3D. The upper bound is bigger than or equal to 301/32 in 2D and 68/3 in 3D.

In dimension 2, we expect every Henneberg step to increase the Laman number by at least a factor of two. As mentioned above, this is still open for steps of type 2c.

Conjecture 4. For a Laman graph *G* with *n* vertices we have Lam_2_(G) ⩾ 2^n − 2^.

In dimension 3, this does definitely not hold any more, since the first line in Table 5 gives a counterexample. It would be interesting to know whether there is a lower bound on the Laman number in 3D.

Another direction of research is the study of real realizations, i.e., by considering labelings λ whose values are in R and embeddings into $R^{d}$. In the 2D case, it is known that the ratio between the number of real and complex realizations can be arbitrarily close to 0, by exhibiting a particular graph (of eight vertices and Laman number 90), which provably cannot have as many real realizations as complex ones [Jackson and Owen 18], and by gluing this graph arbitrarily often together.

Open Problem 5. Let *R*_2_(*G*) be the maximal (finite) number of different real realizations in $R^{2}$ of a Laman graph *G* = (*V*, *E*), that can be achieved for some real labeling λ:E→R. Clearly, for a Laman graph *G* that is constructible by using only Henneberg steps of type 1, we have *R*_2_(*G*) = Lam_2_(G). But what can we say about the sequence $\left(\varphi_{n}\right){}_{n⩾2}$ of quotients

$$\varphi_{n}:=\frac{R_{2}\left(G_{n,\max}\right)}{M_{2}\left(n\right)},$$

i.e., we are asking about the gap between real and complex realizations for graphs with maximal Laman number. From [Emiris and Moroz 11] we know that ϕ_*n*_ = 1 for *n* ⩽ 7, but does ϕ_*n*_ = 1 hold for all *n*? Probably not. Do we have lim_*n* → ∞_ϕ_*n*_ = 0, or does this limit approach a nonzero constant? Does the limit exist at all?

Similar questions can be posed for the three-dimensional case, where much less is known. A first step into this direction would be to answer the following question:

Open Problem 6. Find a Geiringer graph that cannot have as many real realizations as complex realizations.

## Appendix—Graph encodings

In this[Table t0008 t0009 t0010 t0011 t0012 t0013 t0014 t0015 t0016 t0017 t0018]section, we present details on our graph encodings and collect the encodings of the graphs explaining the results and observations in the main part.

We represent a graph by the integer that is obtained by flattening the upper right triangle of its adjacency matrix and interpreting this binary sequence as an integer. Note that the adjacency matrix will always have zeros on the main diagonal, and hence we consider only entries above the main diagonal.

Note, that isomorphic graphs might be represented by different numbers in this way. Hence, for our computations we used some normal form, which is not necessary to explain in detail here. The conversion from a number to a graph does not depend on this normal form.
